# Androgen Deprivation Therapy for Prostate Cancer: Focus on Cognitive Function and Mood

**DOI:** 10.3390/medicina60010077

**Published:** 2023-12-30

**Authors:** Allison B. Reiss, Shelly Gulkarov, Aaron Pinkhasov, Katie M. Sheehan, Ankita Srivastava, Joshua De Leon, Aaron E. Katz

**Affiliations:** 1 Department of Medicine and Biomedical Research Institute, NYU Grossman Long Island School of Medicine, Mineola, NY 11501, USA; shellygulkarov@mail.adelphi.edu (S.G.); katie.sheehan2@nyulangone.org (K.M.S.); ankita.srivastava@nyulangone.org (A.S.); joshua.deleon@nyulangone.org (J.D.L.); 2 Department of Psychiatry, NYU Grossman Long Island School of Medicine, Mineola, NY 11501, USA; aron.pinkhasov@nyulangone.org; 3 Department of Urology, NYU Grossman Long Island School of Medicine, Mineola, NY 11501, USA; aaron.katz@nyulangone.org

**Keywords:** androgen deprivation therapy, cognitive function, prostate cancer, hormonal therapies, management strategies, testosterone

## Abstract

Prostate cancer is the second leading cause of cancer death in men in the United States. Androgen deprivation therapy (ADT) is currently the primary treatment for metastatic prostate cancer, and some studies have shown that the use of anti-androgen drugs is related to a reduction in cognitive function, mood changes, diminished quality of life, dementia, and possibly Alzheimer’s disease. ADT has potential physiological effects such as a reduction in white matter integrity and a negative impact on hypothalamic functions due to the lowering of testosterone levels or the blockade of downstream androgen receptor signaling by first- and second-generation anti-androgen drugs. A comparative analysis of prostate cancer patients undergoing ADT and Alzheimer patients identified over 30 shared genes, illustrating common ground for the mechanistic underpinning of the symptomatology. The purpose of this review was to investigate the effects of ADT on cognitive function, mood, and quality of life, as well as to analyze the relationship between ADT and Alzheimer’s disease. The evaluation of prostate cancer patient cognitive ability via neurocognitive testing is described. Future studies should further explore the connection among cognitive deficits, mood disturbances, and the physiological changes that occur when hormonal balance is altered.

## 1. Introduction

The treatment for hormone-sensitive metastatic prostate cancer commonly entails the use of androgen deprivation therapy (ADT), which, by suppressing or blocking testosterone, deprives the tumor of a key factor driving its growth [1,2,3]. Since approximately 11.6% of males will be affected by prostate cancer in their lifetime, the number of men undergoing this treatment is large and growing [4,5].

Since testosterone plays an important role in cognition and mood, ADT can impact these key characteristics and, therefore, degrade quality of life [6]. These side effects of ADT pose a challenge, particularly because they may reduce adherence to life-prolonging medical therapies [7]. This is a conundrum for the healthcare provider and patients because multiple cooperative studies including randomized clinical trials have shown the efficacy of hormonal therapy in patients with metastatic disease to improve overall survival and decrease skeletal-related events [8,9]. This review examined the scope of consequences brought about when ADT reduces clarity of thought and dampens mood, looks into the physiological changes observed in the brain with ADT, and addresses the knowledge gap in the etiology and mitigation of these symptoms.

## 2. ADT Materials and Methods

This narrative review article was developed using PubMed, Scopus, and Google Scholar. These scientific databases were searched from inception to November 2023 using the terms “prostate cancer”, “androgen deprivation therapy”, “hormone therapy”, “cognition”, “cognitive function”, “dementia”, “mood”, “anxiety”, “depression”, “adverse effects”, “hot flashes/hot flushes”, “quality of life” and “Alzheimer’s disease”. Boolean operators, such as AND/OR, were employed strategically for the effective amalgamation of the search terms. Papers, including original articles and reviews, written in the English language were screened by the authors to retrieve results relevant to the topic of this review based on article novelty, quality, and clinical relevance.

## 3. ADT as Treatment for Metastatic Prostate Cancer

### 3.1. Indications for ADT

ADT is the primary treatment for metastatic prostate cancer, with the benefit of improved survival [10,11,12]. It may also be used short term in patients with localized disease considered at risk for progression [13].

### 3.2. ADT Medications and Their Mechanism of Action

Androgens, secreted primarily by the testicles in men, behave as neuromodulators by activating neuronal androgen receptors. The activation of these receptors then drives changes in gene transcription that affect brain function [14,15]. Androgens support optimal cognitive function, specifically memory [16,17,18].

**Figure 1:**
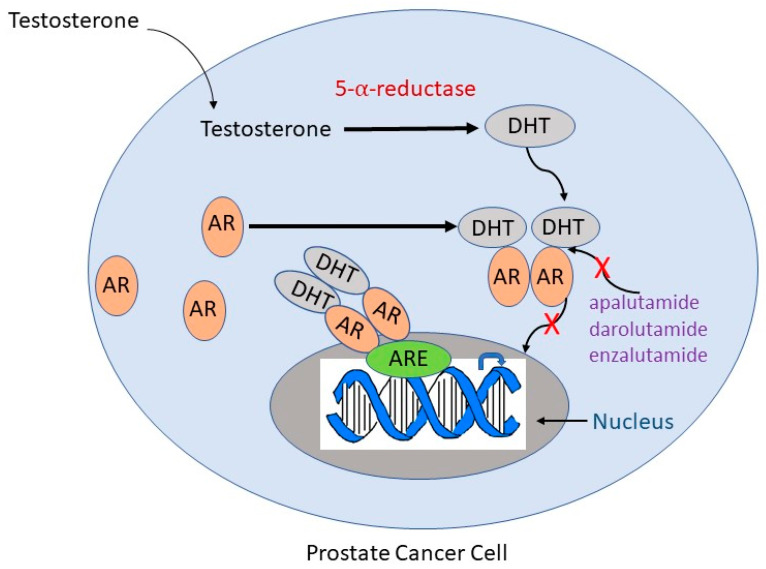
Mechanisms of action of anti-androgen drugs frequently used in ADT. Testosterone is converted to DHT by 5-α-reductase. DHT binds to the AR, causing a conformational change in the receptor that leads to its homodimerization and translocation to the nucleus. In the nucleus, the AR binds to the ARE and acts as a transcription factor to signal downstream targets. Second-generation anti-androgens such as apalutamide, darolutamide, and enzalutamide competitively suppress binding of androgens to the AR (indicated by a red “X”) and inhibit AR translocation to the nucleus (indicated by a red “X”). DHT, dihydrotestosterone; AR, androgen receptor; ARE, androgen response element.

**Figure 2:**
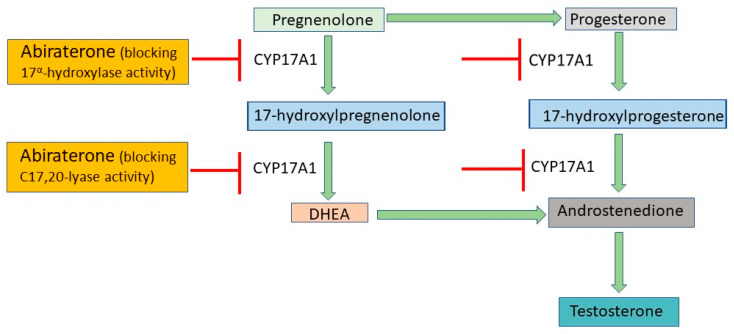
Mechanisms of action of abiraterone. Abiraterone blocks the activity of CYP17A1, a key enzyme in the production of testosterone. By inhibiting CYP17A1, it prevents conversion of pregnenolone to DHEA and progesterone to androstenedione, resulting in decreased testosterone biosynthesis. CYP17A1, cytochrome P450, family 17, subfamily A, polypeptide 1; DHEA, dehydroepiandrosterone.

ADT utilizes androgen receptor antagonists and second-generation anti-androgens to prevent the translocation of androgen receptors to the nucleus [19]. First-generation anti-androgens such as flutamide and nialamide solely prevent the translocation of androgen receptors to the nucleus and downstream signaling, while second-generation anti-androgens such as enzalutamide and abiraterone have their own mechanisms of action [20]. Apalutamide, darolutamide, and enzalutamide act as anti-androgens by inhibiting both androgen binding to its receptor and the translocation of the receptor from cytoplasm to nucleus and have much greater pharmacologic potency than first-generation drugs (Figure 1) [21]. They also impede the binding of the androgen receptor to androgen response elements on DNA, thus inhibiting DNA transactivation. Abiraterone is an irreversible inhibitor of the enzyme cytochrome P17 (CYP17), which has both 17α-hydroxylase and C17,20-lyase activity and catalyzes multiple steps in androgen biosynthesis (Figure 2) [22]. Either abiraterone or enzalutamide as second-generation anti-androgens can reduce tumor burden, prolong life, and relieve symptoms, but resistance to the drugs eventually allows disease progression [23,24].

### 3.3. ADT Risks and Side Effects

#### 3.3.1. Overview of Side Effects

ADT comes with a variety of side effects and increased risks [25]. Androgen signaling is essential for maintaining homeostasis in multiple organ systems, and ADT treatment relies on disrupting this pathway, leading inevitably to a number of negative side effects. Later in this review, we will focus on the cognitive, mood, and tiredness issues related to ADT [26,27]. In addition, quality of life is affected by ADT due to other common consequences of androgen deficit. These can include hot flushes, sexual dysfunction, bone loss, and heightened cardiovascular risk [28,29,30,31].

#### 3.3.2. Hot Flushes

Hot flushes, also referred to as hot flashes, are characterized by a sudden and intense sensation of heat, particularly in the face, throat, and extremities. The body reacts to this perceived temperature rise, causing cutaneous vasodilation, excessive sweating, rapid heartbeat, chills, night sweats, and feelings of anxiety. These physiological responses are often accompanied by redness in the face and neck [32]. These episodes can be triggered by hormonal changes, specific medications, or underlying medical conditions. The intensity and duration of hot flushes can differ from one person to another, and they may affect daily routines and sleep patterns [33]. Sleep disturbance brought on by hot flushes can adversely affect cognitive function [34].

In a randomized, double-blind study involving 208 patients with androgen-dependent prostate cancer, the quality of life of participants was assessed after a 3- to 4-month run-in phase with ADT followed by the randomization of sipuleucel-T cellular immunotherapy or control at a 2:1 ratio. During the first week, the most frequently observed symptoms were hot flashes/sweats (87.3%), reduced sexual desire (66.1%), and reduced sexual function (50.9%). However, after the discontinuation of ADT during the randomized phase, the occurrence of these symptoms decreased [35]. In comparison, Kaplan et al. found that hot flashes were reported in only 13 out of 62 patients receiving enzalutamide and radiotherapy. Those who exhibited hot flushes reported very mild symptoms [36]. In a retrospective, medical chart review study, Hussain et al. found that, in a subset of prostate cancer patients treated with apalutamide or enzalutamide and experiencing adverse events, the incidence of hot flushes was 13.9% [37]. In other studies, hot flushes were typically reported in about 50% of men undergoing ADT and were often considered the most bothersome side effect [38]. The mechanism of hot flushes in men undergoing ADT remains unclear. It is suggested that, following GnRH agonists, there is a rapid decline in serum luteinizing hormone and follicle-stimulating hormone levels, triggering the release of hypothalamic catecholamines. These catecholamines may potentially overwhelm the thermoregulation center in the hypothalamus, resulting in a dysregulated response to temperature changes [39]. The body’s thermoregulation system appears to be influenced by androgens. When individuals experience a significant reduction in hormone levels, as is the case with men taking androgen antagonists, the body responds in a manner that induces hot flushes.

Several treatment strategies have been applied to alleviate the intensity of hot flushes. Small doses of estrogen aim to restore the balance of hormone levels and mitigate symptoms of ADT [29,40]. Adverse effects such as gynecomastia are the downside of this treatment [41]. Steroidal progestins, such as megestrol acetate, cyproterone acetate, and medroxyprogesterone, are also associated with the reduction in hot flushes [42]. However, steroidal progestins are linked to a wide range of side effects, including nausea, weight gain, muscle spasms, depression, insomnia, and headaches [41,43]. Clonidine, an alpha-2 receptor agonist, may provide relief of hot flushes by impacting the thermoregulatory center, but its effectiveness is uncertain [41,42,43]. Non-hormonal treatments may also include selective serotonin reuptake inhibitors (SSRIs) and antidepressants may be helpful [42].

#### 3.3.3. Sexual Dysfunction

Men who have been prescribed and take androgen antagonists experience a wide array of sexual side effects. Understanding the primary factors impacting sexual function in men is challenging, given the multitude of psychological, environmental, and physiological aspects that influence sexual performance. Nevertheless, well-established evidence demonstrates a significant role for testosterone in influencing sexual drive and, as a result, low testosterone levels are linked to low libido and impaired erectile and orgasmic functions [44,45].

The corpora cavernosa, two cylindrical structures running along the length of the penis and responsible for erection, contain androgen receptors that govern the key biochemical pathways essential for achieving an erection [46]. Achieving an erection is a multifaceted physiological event that begins when the nervous system releases nitric oxide (NO) and other neuroendocrine factors, which promote the relaxation of smooth muscle cells in the penile arteries and corpora cavernosa, causing increased blood flow to the penis. The veins that typically drain blood from the penis become compressed, aiding in maintaining the erection and ensuring turgidity. The initial release of NO is partly facilitated by testosterone [47]. Reduced testosterone can induce endothelial dysfunction, a condition where the endothelium, the inner lining of blood vessels, loses its ability to regulate vascular tone and function properly. Low testosterone impacts NO levels by reducing NO synthase expression and activity and increasing asymmetric dimethylarginine (ADMA), an endogenous competitive inhibitor of NO formation. Furthermore, testosterone may influence the endothelial repair system by modulating the proliferation and migration of endothelial progenitor cells [48]. Consequently, low levels of testosterone can make it increasingly challenging to initiate and sustain an erection. While the exact mechanism of how testosterone impacts libido is unclear, the association between reduced libido and low testosterone levels has been well documented [49]. Sexual dysfunction is a potential outcome across almost all treatment options for prostate cancer, but ADT may exacerbate these symptoms even further. These findings have raised concerns about the effects of ADT, not only on the health-related quality of life of patients, but also on the overall well-being of their intimate relationships [50]. The intensity of these symptoms can be so debilitating and disturbing that it is associated with a lack of adherence to treatment, despite a heightened risk of relapse or mortality [51]. When undergoing ADT, it becomes evident that the removal of testosterone from the body not only impairs erectile and orgasmic functions but also diminishes the desire to participate in sexual activities.

#### 3.3.4. Bone Density

ADT is associated with reduced bone mineral density (BMD) and an increased risk of fractures [52,53]. Testosterone plays a crucial role in maintaining the strength and density of bones by regulating the balance between bone formation and resorption. ADT decreases testosterone levels and disrupts this balance, leading to bone loss with skeletal fragility. Maintaining the proper balance of receptor activator of nuclear factor k-B ligand (RANKL) is crucial for the equilibrium between bone formation by osteoblasts and bone resorption by osteoclasts. Increased RANKL levels cause increased bone resorption, leading to a decrease in BMD and the development of osteoporosis [54,55,56].

BMD decline with ADT is not preventable, but adequate calcium and vitamin D intakes are important. Osteoporosis risk factors that can be modified include avoiding tobacco and limiting alcohol consumption [53]. Pharmacological approaches aimed at preserving or enhancing bone health include medications that inhibit bone resorption, such as bisphosphonates and denosumab [57].

#### 3.3.5. Cardiovascular Effects of ADT

ADT substantially heightens cardiovascular event risk as well as the risk for hypertension and arrhythmia [6,58,59]. ADT has been associated with an increased risk of developing metabolic conditions that contribute to cardiovascular risk such as insulin resistance, dyslipidemia, diabetes, and metabolic syndrome [60,61]. Of considerable significance, cardiovascular disease stands as the primary cause of mortality among individuals with prostate cancer [62]. Reducing the cardiovascular toxicity of ADT is generally approached by controlling modifiable risk factors such as blood pressure and lipid profile while encouraging a healthy diet, physical activity, and the avoidance of tobacco and excess alcohol consumption [63]. The etiology of cardiovascular disease in men with prostate cancer continues to be a subject of ongoing research [64,65].

## 4. Testosterone Impact on Cognition, Mood, and Energy

Testosterone plays a pivotal role in mood, behavior, cognition, and quality of life in men at every age [7,66,67]. Androgens, secreted primarily by the testicles in men, behave as neuromodulators by activating neuronal androgen receptors. Androgen receptors are present throughout the brain [68]. The activation of these receptors then drives changes in gene transcription that affect brain function [14,15]. Androgens support optimal cognitive function, specifically memory [16,17,69]. The neuroprotective role of androgens such as testosterone and its metabolite dihydrotestosterone (DHT) is especially important against neurogenerative diseases, such as AD, and protects neurons from inflammatory damage induced by activated microglia [70].

Testosterone has neuroprotective effects on the brain, and studies have shown the impact of testosterone on cognition [71]. Hypogonadal men demonstrate an impairment in spatial abilities, memory, and attention, and the administration of testosterone and DHT gel improved cognition [72,73].

In a retrospective analysis, Giannos et al. found a positive relationship between bioavailable serum testosterone and the Digit Symbol Substitution Test, an indicator of processing speed, in males aged 60 and above [74]. A study from Japan of older men found that higher salivary testosterone concentration measured by immunoassay was associated with better global cognitive function [75]. In a study of prostate cancer patients from Poland, higher testosterone levels in those who had received ADT were associated with better cognitive function based on neuropsychological assessment [76]. These findings support the influence of androgens such as testosterone on cognitive function and the potential of cognitive disruption with anti-androgen treatment.

In addition, various animal studies also support the impact of testosterone on hippocampal synaptic plasticity and, hence, memory and learning [77,78,79,80].

Men with lower testosterone levels are at a higher risk for developing depressive illnesses [81]. Male hypogonadism, a condition in which the body does not produce enough testosterone, causes a constellation of symptoms that includes dysphoria, irritability, fatigue, decreased libido, and decreased concentration, all of which are shared with depressive disorders [82,83,84,85]. The evident correlation between depressive symptoms and low levels of testosterone points to an antidepressant effect of testosterone in males [86,87,88].

Overall, testosterone is shown to improve cognition and mood in males because of its role as a neuromodulator that increases synaptic plasticity and the symptomatic effects seen in hypogonadal men deprived of the hormone.

## 5. ADT, Cognition, and Mood

### 5.1. The Controversy

Cognition and mood are closely interrelated such that more severe symptoms of depression can exacerbate cognitive decline in older persons [89,90].

There are conflicting data in the literature about the relationship between ADT and cognitive function decline, but recent evidence supports these changes, particularly in language skills and processing speed [91,92]. A meta-analysis of 12 studies comprising 13,524 participants examined the cognitive effects of second-generation anti-androgen therapy (abiraterone, apalutamide, darolutamide, and enzalutamide) in men treated for prostate cancer and found a statistically significant increase in the risk of cognitive toxic effects such as disturbed attention, memory impairment, cognitive disorder, and amnesia with treatment compared to placebo [93]. The effect of ADT on mood is seen in both objective measures and by self-report of patients [94,95]. A clinical trial ongoing at this time will help to determine whether testosterone replacement can improve cognition and mood in prostate cancer survivors deficient in the hormone [96]. As outlined in Section 3, many studies have shown that a reduction in overall testosterone level, which is the endpoint of these medications, does lead to mood changes and cognitive impairment, and this has been well documented. In our efforts to improve the treatment of prostate cancer, it is important to investigate factors that may explain why some patients develop cognitive decline and depression on ADT and others do not.

### 5.2. Neurocognitive Tests

In assessing cognitive health in prostate cancer patients, tools used are generally the traditional, reliable, and validated tests (Table 1) [97]. A comprehensive battery of testing may not be practical in the oncology office setting. One cognitive screening tool commonly used is the Montreal Cognitive Assessment (MoCA), which is a 30-point test with very good sensitivity for mild cognitive impairment [98,99,100,101]. A higher score denotes better cognitive performance. A MoCA score more than 1.5 standard deviations below age- and education-specific normative values is categorized as probable cognitive impairment [102,103].

Another cognitive workup used is the Mini-Mental State Examination (MMSE), which is a brief assessment to test cognitive impairment by testing orientation, memory, attention, and verbal and written ability [104,105]. A maximum MMSE score is 30 points, and cutoffs for determining impairment vary in the literature, with mild impairment generally falling in the range of 25–27 points [106,107]. An additional cognitive test is the Mini-Cog, which is a brief assessment that is easy to administer as it consists of a delayed three-word recall task and a clock-drawing task [108,109].

The N-back test is a test of working memory that is useful for experimental research on cognition and aspects of intelligence such as fluid intelligence [110,111]. The participant is presented with a stimulus in a sequence and the participant is then asked to match the current stimulus to a previous one or to determine whether the current stimulus is the same as the stimulus “n” items ago or “n” positions earlier in the sequence [112].

The CogState Brief Battery (CBB) test is a brief, 10-min, computer-administered exam that includes four cognitive tasks including psychomotor function, attention, working memory, and memory [113,114]. Each task has simple yes or no answers, and the simplicity of the test has made it well validated in neurocognitive studies. This test is noted for its sensitivity and validated in use for patients with cancers, MCI, and AD [115].

Various sources have illustrated that longer cognitive tests, around 45 min, do not consistently provide more accurate results than these brief workups, and, therefore, these brief examinations are more practical and likely to be applied in a clinical setting [116,117].

**Table 1:** Neurocognitive tests commonly used to screen for and assess cognitive deficits.

Cognitive Test	Description	Domains Tested	Scoring Method	References
MoCA	A brief cognitive screening tool with high sensitivity and specificity for detecting MCI.	(1) Memory, (2) executive functioning, (3) attention, (4) language, (5) visuospatial, and (6) orientation	30-point total. Lower score indicates poorer performance	[98,99,100,101]
MMSE	Well-validated assessment of cognitive function. Takes approximately 10–15 min to administer.	(1) Orientation, (2) immediate memory, (3) attention/concentration, (4) delayed recall, (5) language	30-point total. Lower score indicates poorer performance	[104,105,106,107]
Mini-Cog	Cognitive screening tool that takes about 3 min to administer. Used in various healthcare settings. Has 2 components: 3-word recall and clock drawing.	(1) Cognitive function, (2) memory, (3) language comprehension, (4) visual-motor skills, (5) executive function	Recall graded on a scale of 1 to 3. Clock draw graded 0 or 2. Total score of 2 or below indicates a positive dementia screen. Total score of 3 or above is negative	[109]
N-back test	Test of working memory capacity. Participants required to integrate and recall stimulus sequences presented in a specific order.	Working memory with either visual or auditory presentation	Score calculated by dividing mean correct response times by proportion of hits for each participant and for each level of N-back	[110,111,112]
CogState Brief Battery	A brief computerized test with 4 tasks and simple “yes” or “no” answers	Psychomotor function, attention, working memory, and memory	For the psychomotor, attention, and working memory tasks, scores are the log10 transformed mean response times of correct trials. For short-term memory task, scores are the arcsine of the square root of the proportion of correct responses	[114]

MoCA, Montreal Cognitive Assessment; MCI, mild cognitive impairment; MMSE, Mini-Mental State Examination.

### 5.3. ADT Impact on Cognition

There is a good deal of controversy surrounding the potential for ADT to impair cognitive function [118]. Various studies have demonstrated a negative impact of ADT on aspects of cognition that include memory, attention, and executive function [14,119,120,121]. The duration of ADT exposure may vary, but cognitive assessments are generally carried out at about 1 year after the initiation of ADT [102,122]. It is difficult to directly relate ADT to cognition because of confounding issues. The cognitive domains measured may be affected by insomnia, anxiety, and fatigue brought on by ADT [123]. Garland et al. performed a prospective study with a cohort of 83 ADT recipients with a control group of 92 prostate cancer patients not on ADT [124]. Cognitive function and satisfaction were measured using the Everyday Cognition Scale (ECog), and insomnia was measured with the Insomnia Severity Index. Men with greater depressive symptoms had a stronger association between insomnia severity and worsened cognitive function. The correlation between insomnia and worsened cognition does not necessarily mean causation, as various studies have shown mixed results regarding sleep and cognition [125,126].

A prospective study of a cohort of 366 patients with prostate cancer at the Portuguese Institute of Oncology of Porto used the Montreal Cognitive Assessment (MoCA) for cognitive evaluation at baseline (before treatment) and 1 year later [127]. Treatment consisted of either ADT, radiation therapy, prostatectomy, or combinations of ADT with radiotherapy or chemotherapy. About 5% of subjects had only active surveillance. Cognitive decline was more frequent in the ADT group, which consisted of those receiving ADT with or without other treatments, and the incidence of cognitive impairment was 6.9%. It should be noted that this study was conducted after the COVID-19 pandemic onset, which may have had an influence on the degree of cognitive impairment due to the mandatory quarantine, which limited time spent with nature, associated with worse mental health and insomnia [128].

In a study from the Central Denmark Region of cognitive changes with ADT, Buskbjerg et al. included 37 prostate cancer patients on ADT for a 6-month period along with 27 healthy controls. They found that prostate cancer patients demonstrated reduced testosterone levels and higher rates of decline for 13 out of 15 cognitive outcomes [129]. Three of these outcomes reached statistical significance, relating to verbal memory and visuospatial learning and memory. A meta-analysis of 14 studies also found visuomotor ability to be worse in prostate cancer patients undergoing ADT compared with non-cancer controls [27].

Cherrier et al. performed a comparative whole-brain mapping analysis on nine men with prostate cancer and a rising PSA after primary therapy who were then given ADT (flutamide and leuprolide) for 9 months [130]. The subjects underwent 18F-fluorodeoxyglucose (FDG) positron emission tomography (PET) to assess metabolic changes in the brain before the start of ADT and again after 9 months of treatment. Following 9 months of ADT, decreases in brain metabolism were detected in the posterior cingulate region, cerebellum, and thalamus. Altered glucose brain metabolism corresponded to changes in cognition discerned through a variety of tasks that measured spatial recall, verbal memory, and verbal learning while on ADT. Neuroimaging analysis captured a negative correlation between glucose metabolism in the left inferior parietal lobule and verbal memory tasks. Brain regions impacted by ADT are similar to the brain regions with metabolic decline found in early AD and diabetes, suggesting the possibility of common mechanisms needing further study. Gaynor et al. examined the association between cognitive function and exercise in men over the age of 65 with prostate cancer undergoing ADT [131]. The study utilized the Godin–Shephard Leisure-Time Physical Activity Questionnaire as a self-reporting measure to categorize the frequency of exercise and the neuropsychological battery assessment to assess corresponding performance in cognitive domains. The study enrolled 64 participants and found that exercise positively correlated with performance on tests of memory, attention, and executive function. The authors concluded that exercise could overcome some of the harmful effects of ADT on neurocognitive function. Chaudhary et al. found, in a small sample of men with prostate cancer, that ADT resulted in an isolated decreased accuracy on the 1-back test at 6 months compared to non-ADT controls [132]. This paper will be further discussed in the section “Mechanisms Involved in Effect of ADT on Cognition”. 

### 5.4. ADT Impact on Mood

As noted previously, testosterone is supportive of maintaining mood and acts as an antidepressant [133]. ADT is known to be associated with an increase in depression and anxiety, with some men feeling a loss of masculinity [134,135,136]. Fatigue, depression, irritability, tension, loss of vigor, and anxiety are examples of mood changes noted in men undergoing ADT [137,138,139]. Greater fatigue and depression in these patients were at least partly attributed to ADT, and Cherrier et al. suggested minimizing ADT as much as possible to avoid some of these negative effects [137]. Accompanying depression is a higher risk of suicide, and this was found to be the case in ADT-treated men in a Swedish study where relative rates of suicide were higher in men treated with ADT alone (compared to radiation and/or surgery) and particularly those treated with gonadotropin-releasing hormone analogs compared to anti-androgen monotherapy [140]. 

Nowakowska et al. performed a retrospective cohort study of men receiving either traditional hormone therapy (luteinizing hormone-releasing hormone agonists or antagonists and/or first-generation androgen receptor blockers), no therapy, or second-generation anti-androgen therapy (abiraterone, apalutamide, darolutamide, or enzalutamide) [141]. They found that those who received the more potent second-generation drugs had a large and clinically significant increased risk of depression compared to the other groups.

A study by Tsao et al. using de-identified data from a comprehensive U.S. commercial and Medicare Advantage claims database analyzed a final cohort of 37,388 men with a prostate cancer diagnosis undergoing ADT and found that 10.6% were newly diagnosed with depression or anxiety after starting ADT [142]. They compared this group to a cohort with a prostate cancer diagnosis not undergoing ADT and also to another cohort never diagnosed with prostate cancer. The cohort of men with prostate cancer undergoing ADT had a higher rate of depression and anxiety when compared to the control cohorts. Further, of those who developed depression and anxiety after starting ADT, 47.7% did not receive adequate mental healthcare. The study authors brought attention to the need to improve mental health treatments for men receiving ADT. Finally, a prospective longitudinal observational study from Valencia, Spain, looked at mood and cognitive function in men with prostate cancer at two timepoints: within 6 months of first receiving ADT (luteinizing hormone-releasing hormone analogues) and at follow-up 12 months after the first evaluation [143]. The Geriatric Depression Scale and Athens Insomnia Scale were used to analyze depression and sleep quality, respectively. The study found that men described more sleep disturbances and depressive symptoms at 12 months. Their insomnia scores were significantly worse at the later time and, although they also described more depressive symptoms, the results were not statistically significant in this small group of 33 men.

### 5.5. ADT and Fatigue

Fatigue is a prevalent and distressing side effect experienced during cancer treatment, significantly affecting quality of life. Nelson et al. conducted a prospective longitudinal, observational study to examine fatigue severity in men about to undergo ADT. The participants were assessed at two intervals: 6 months and 12 months after study initiation. At each timepoint, the participants completed the 14-item Fatigue Symptom Inventory (FSI), with a score of greater than 4 being clinically meaningful [144]. Throughout the 12-month follow-up period, the prevalence of clinically meaningful fatigue increased by 20% among patients undergoing ADT. Comparatively, a notably higher proportion of ADT-treated patients than controls reported clinically meaningful fatigue at 6 months (33% vs. 21%) and 12 months (32% vs. 19%). The etiology of treatment-related fatigue has been subject to various hypotheses, but the precise underlying mechanism remains unidentified. One theory is that testosterone protects mitochondrial function and its dearth leaves brain cells vulnerable to oxidative stress and other sources of mitochondrial damage [145,146,147].

## 6. ADT Impact on Quality of Life

ADT affects many aspects of everyday life that can reduce its quality, and among the most prominent is disturbed sleep, which can contribute not only to brain fog but to poor functioning throughout the day [148]. Hot flushes also reduce quality of life, and this may be partly because they can interrupt sleep [149]. Studies show an unclear relationship among ADT, cognition, and quality of life. In a prospective study by Chaudhary et al., 28 individuals with biopsy-proven prostate adenocarcinoma without distant metastases receiving ADT were compared to 38 individuals in the control group not receiving ADT. Quality of life was measured using the Functional Assessment of Cancer Therapy-Prostate (FACT-P) questionnaire, and working memory was assessed by N-back score at baseline and 6-month follow-up. The study did not note any significant differences in quality of life and working memory between the ADT and non-ADT control groups [150]. The short duration and small sample size are factors that may account for a lack of significant effects, and, thus, monitoring for longer than 6 months in an expanded group might detect long-term effects of ADT on cognition and quality of life.

A study from the United Kingdom reported that almost 50% of men living with advanced prostate cancer experience poor quality of life and, not surprisingly, the degree of advancement of the prostate cancer diagnosis correlated with diminution in the quality of life [151]. In addition to insomnia and depressive symptoms, hot flushes induced by ADT may also negatively affect quality of life [152,153,154,155]. Although there are no targeted treatments to mitigate the worsening of quality of life that may accompany ADT, a healthy lifestyle with regular physical activity may be of benefit in reducing fatigue and minimizing the increase in fat mass induced by ADT [156].

The broad spectrum of side effects of ADT, both confirmed and tentative, are summarized in Table 2.

## 7. Possible Mechanisms Involved in Effect of ADT on Cognition

The underlying pathways that lead to cognitive deficits with ADT are not well understood and this is an important knowledge gap with ramifications for a large number of people living with prostate cancer. This section will present a number of proposed mechanisms that may be involved. Cognitive changes may be brought about by a combination of these rather than by any one specifically.

### 7.1. Changes to the Hypothalamus and Corpus Callosum

Changes in the hypothalamus have been a focus of interest in the realm of cognition and ADT [130,157]. The importance of the hypothalamus for memory and emotion makes the physiological changes observed in the hypothalamus as a result of ADT significant [158,159]. A small longitudinal study with functional brain imaging from the Medical Oncology and Urology Clinics at the West Haven VA Connecticut Healthcare System enrolled individuals with biopsy-proven prostate cancer who had never been treated with ADT for evaluation at baseline and 6 months. Of this group, 25 patients who underwent ADT and 30 control group patients who did not receive ADT completed the study. A correlation was found between elevated hypothalamus-right mid-cingulate cortex and precentral gyrus resting state functional connectivity in those receiving ADT compared to those not given ADT [157]. Although the exact mechanism of this association is unclear, future studies with longer ADT monitoring periods and other cognitive/behavioral markers are needed to evaluate the importance of this correlation. The study authors suggested that enhanced hypothalamic functional connectivity may be a compensatory mechanism to counterbalance the detrimental effects of ADT on cognition and memory. Other studies, specifically on mood, noted a negative association with the hypothalamus and brainstem for depression, anxiety, and aggression [130].

Chaudhury et al. published another study from their patient group at the Medical Oncology and Urology Clinics at the West Haven VA Connecticut Healthcare System in which they looked at changes in fractional anisotropy, a diffusion tensor imaging metric indicative of microstructural changes in white matter [132]. They evaluated the cognitive effects of ADT in men with prostate cancer using the MoCA and magnetic resonance imaging performed at baseline and at a 6-month follow-up. Of 33 men with prostate cancer evaluated at both timepoints, 16 received ADT and 17 received no ADT and served as controls. One ADT and two control subjects were excluded because the quality of MRI scans was inadequate, leaving 15 ADT and 15 non-ADT controls for comparison. The study found that fractional anisotropy was reduced in the corpus callosum with ADT at follow-up. The corpus callosum is the largest white matter structure in the brain. It connects cortical regions of both hemispheres, and structural alterations in this region may account for a deficit in cognitive-motor processing [160,161,162]. The corona radiate, an anatomic linkage that supports cognitive and motor systems, also showed a reduced fractional anisotropy at follow-up. Patients with fewer changes in brain structures such as the corpus callosum, superior and posterior corona radiata, and anterior thalamic radiation demonstrated less impaired cognitive-motor processing.

### 7.2. Potential Association between Cytokine Release and Cognitive Deficits

Cytokines are low-molecular-weight cell signaling proteins released by numerous cell types that can promote the survival and proliferation of prostate cancer cells [163]. Cytokines may play a role in causing cognitive changes during ADT [164]. A number of cytokines increase in quantity with metastasis and resistance to ADT, the most prominent of which is interleukin (IL)-6 [165]. IL-6 levels in humans have been proposed as an indicator of overall cognitive health [166,167,168]. Increases in circulating IL-6 with ADT have been linked to symptoms of fatigue and frailty in prostate cancer patients [169,170].

Verma et al. performed a bioinformatic analysis, looking at transcriptomic data from prostate cancer patients receiving ADT and those who were not given ADT; they found increased inflammatory cytokine signaling after ADT and they linked these cytokines to brain deficits [171]. Among the cytokines increased in ADT patients were leukemia inhibitory factor receptor (LIFR, a receptor for the IL-6-like cytokine LIF), IL-1 receptor antagonist, IL-10, chemokine ligand (CCL)2, a chemokine known to contribute to prostate cancer resistance, and the IL-17RA receptor [172,173].

Exposing cultured brain glial M059K cells to the second-generation anti-androgen enzalutamide raises the mRNA expression level of the cytokines IL-6, IL-17A, IL-1 receptor antagonist, IL-10, CCL2, and LIF and its receptor LIFR [173,174,175]. IL-17A has been reported to be involved in neurodegenerative diseases such as Alzheimer’s disease, Parkinson’s disease, and multiple sclerosis [176]. Li et al. showed that serum and CSF levels of IL-17A increase with age in humans and performed accompanying murine studies, showing a role for this cytokine in neuroinflammation and microglial activation and a possible effect on BBB integrity and cognitive aging [177].

Much more research is necessary to determine whether there is any causal relationship between cytokines and neurological changes in persons with prostate cancer on ADT.

## 8. Is There a Relationship between ADT and Alzheimer’s Disease?

Alzheimer’s disease (AD) is the most common cause of dementia and is more prevalent in males than females [178]. In the brain, AD is manifested pathologically by the accumulation of proteinaceous extracellular plaques consisting of beta-amyloid and intracellular neurofibrillary tangles composed of hyperphosphorylated tau [179]. Steroid hormones, specifically testosterone, are known to support cognitive function. As men age, cognitive function tends to decline, as do testosterone levels [180]. Low testosterone levels are considered a risk factor for the development of memory loss and AD [181,182]. The reduction in serum testosterone due to the use of ADT medications may be associated with AD [183]. Klusters et al. and Yeung et al. each found an association between higher androgen exposure and decreased AD risk among men of European ancestry [184,185]. Multiple groups have found that ADT increases AD risk [186,187]. There are also publications showing increased dementia risk with ADT that do not distinguish AD from non-AD dementia [188].

There is also a body of research that finds no link between ADT and AD [189,190]. A systematic review and meta-analysis of 50,541 individuals with prostate cancer showed a statistically significant positive correlation for dementia with ADT but no statistically significant association between ADT and AD [191]. A meta-analysis using single nucleotide polymorphism (SNP) data for rs429358 and rs7412 to determine ApoE genotypes looked at 13,203 men with prostate cancer, with 132 subjects receiving ADT and 13,017 subjects not exposed to ADT. The results indicated that ADT was not associated with AD [192]. Another study evaluated the effect of ADT on the incidence of dementia using the Korean Central Cancer Registry with 9880 prostate cancer patients in the ADT group and 51,206 in the non-ADT group. From this database, 9.3% developed dementia, including AD and Parkinson’s disease, during the study period from 2006 to 2013 [193]. After multivariate analysis, there was no statistically significant correlation between dementia incidence with ADT; rather, older age, cerebrovascular disease, and a higher Charlson Comorbidity Index (a validated method of estimating the risk of death from comorbid condition) score were risk factors for dementia. Bringing more uncertainty to the field, a recent retrospective cohort study of men with prostate cancer found that ADT was associated with lower AD risk [194].

As noted earlier, IL-6 and other inflammatory cytokines are implicated in treatment resistance of prostate cancer and are affected by ADT [195]. IL-6 may contribute to cognitive dysfunction [196]. The bioinformatics study by Verma et al., discussed previously in the section on cytokines, also compared an AD patient cohort with metastatic prostate cancer patients receiving neoadjuvant ADT [171]. They performed gene expression profiling using RNA sequencing data and found 33 common genes that were differentially expressed in both prostate cancer patients undergoing ADT and AD patients. Notably, IL-6 was highly expressed in both ADT and AD groups. Testosterone deficiency relates to an increase in pro-inflammatory cytokines [197,198]. The association of inflammatory cytokines such as IL-6 and cognitive impairment in both prostate cancer patients undergoing ADT and AD patients merits further scrutiny with a focus on both cognitive measures and physiological effects.

A number of animal studies showed the influence of androgens on AD pathophysiology [199,200]. In an AD rat model induced by intra-hippocampal injections of oligomeric beta-amyloid peptide 1–42, cognitive performance was impaired. Testosterone treatment of these AD rats resulted in better cognitive performance, but, when flutamide, a nonsteroidal androgen receptor antagonist, was used to block androgen effects, the benefits of testosterone were nullified [201,202]. In the same study, immunohistochemistry and Western blot analysis showed that the testosterone treatment alone decreased expression of beta-amyloid 1–42 protein and increased synaptophysin, while treatment with both testosterone and flutamide blocked all testosterone-mediated effects. These data suggest that testosterone acts via androgen receptors to remove beta-amyloid, leading to enhanced synaptic plasticity. Synaptophysin is one of several biomarkers for synaptic damage in AD, and anti-androgen drugs seem to damage synaptic connectivity [203]. Thus, second-generation anti-androgens such as flutamide may interfere with the removal of beta-amyloid.

Dysfunction of the hypothalamic–pituitary–gonadal axis has been postulated to participate in AD pathogenesis through glucocorticoid over-secretion and the resulting neurotoxicity, oxidative stress, and inflammation [204,205,206,207]. In prostate cancer, ADT blocks the hypothalamic–pituitary–gonadal axis, and studies have shown that interfering with this axis may cause not only cognitive decline but physiological changes in the hypothalamus and white matter structures of the brain [130,132,157,208].

At this time, although the link between AD and ADT may be tenuous, dysregulation of the hypothalamic–pituitary–gonadal axis in ADT-treated prostate cancer and AD may be a clue to pursue in our efforts to understand any possible commonalities of mechanisms. Gender-based differences in AD risk combined with the likelihood that pathways are shared amongst dementias lead us to surmise that revealing the mechanisms and genetic factors underlying ADT-related changes in cognition could move dementia research forward and lead to insights into AD pathophysiology [209,210]. Further, the use of ADT in persons vulnerable to develop AD could accelerate neurodegenerative processes and bring forth the appearance of symptoms of cognitive decline [211].

## 9. Limitations

This review provides a synopsis of our current knowledge of the cognitive and mood changes associated with ADT treatment of prostate cancer. Several limitations are noted, particularly related to the small number of studies published using a formal neurocognitive evaluation to compare pre-ADT and post-ADT effects. In addition, the field of ADT is moving very fast and newer drugs may differ from older-generation options. However, there is not enough data to compare head to head the different types of therapy. Another issue is the inescapable intertwining of symptoms that may be prostate cancer related versus those that are ADT related. For example, depressed mood may be due to the reality of having cancer, quality of life changes such as loss of sexual function, the ADT itself, or a combination. Self-reporting is a useful tool for gathering data in assessments of sleep, exercise, and other variables but is inherently prone to measurement error and bias [212]. Finally, demonstrating an association between ADT and mood or cognitive changes is not sufficient to conclude causality or infer mechanism.

## 10. Conclusions

After a review of the literature, it became evident that engaging in detailed discussions with patients regarding the potential side effects of ADT and its implications on their overall quality of life is imperative. This involves establishing practical expectations for the mitigation of these effects. However, it should be noted that a significant number of these adverse effects continues to be the subject of ongoing research. Testosterone has a neuroprotective role, which is especially important in the context of neurodegenerative conditions such as Alzheimer’s disease. The question of whether ADT contributes to the development of Alzheimer’s disease remains contentious, as conflicting research findings have emerged. Some studies propose that testosterone potentially influences cognitive functions like memory and learning. Furthermore, there is an indication that anti-androgens promote inflammation, impede the clearance of beta-amyloid, and lead to a reduction in brain metabolism. There is a necessity for further research to compare the hypothalamic–pituitary–gonadal axis in individuals undergoing ADT for prostate cancer with those not undergoing ADT, with extended monitoring periods for ADT effects, along with additional cognitive and behavioral indicators.

## Figures and Tables

**Table 2:** Summary of side effects of androgen deprivation therapy.

Category of Side Effect	Symptoms	References
Cognition	* Memory impairment * Disturbed attention * Deficits in information processing Altered glucose metabolism in brain	[27,93,129,130,131,132]
Mood disturbances	Depression Anxiety Irritability Fatigue Sleep disturbances Tension Loss of vigor	[133,134,135,136,137,138,139]
Vasomotor	Cutaneous vasodilation (hot flushes) Excessive sweating Rapid heartbeat Feelings of anxiety Reduced sexual desire and function Sensation of heat in face, throat	[32,33,34,35,36,37,38]
Sexual dysfunction	Low libido Impaired erectile and orgasmic functions	[44,45,46,47,48,49]
Bone density	Reduced bone mineral density Increased risk of fractures Bone loss with skeletal fragility	[52,53,54,55,56]
Cardiovascular	Hypertension Arrhythmia Elevated risk of cardiovascular event Insulin resistance Dyslipidemia Metabolic syndrome	[6,58,59,60,61,62]

* Putative, not yet firmly established.
